# Are There Heterogeneous Impacts of Air Pollution on Mental Health?

**DOI:** 10.3389/fpubh.2021.780022

**Published:** 2021-11-19

**Authors:** Qingqing Hu, Yanhong Feng, Mark Xu

**Affiliations:** ^1^School of International Studies, Hunan Institute of Technology, Hengyang, China; ^2^School of Economics and Statistics, Guangzhou University, Guangzhou, China; ^3^Portsmouth Business School, University of Portsmouth, Portsmouth, United Kingdom

**Keywords:** air pollution, mental health, heterogeneous impact, mediating effect, income

## Abstract

Many studies reveal that air pollution is related to mental health. However, the level of impact and the regulatory mechanism of air pollution on different types of mental health are unknown. This paper examines the heterogeneous impact and mediating mechanisms of air pollution on mental health based on data of 51 countries from 2010 to 2017 by using panel Tobit random effect model, mediating effect model, and bootstrap test. The findings show that, firstly, there is heterogeneous impact of air pollution on different types of mental health. Specifically, air pollution has a significant positive impact on depression; and the impacts on happiness and anxiety are closely related to income level. Secondly, the heterogeneous impact of air pollution on mental health is contingent on income levels. Thirdly, the heterogeneous impacts under different income levels are exacerbated by different levels of education and population density. Lastly, the mediating effect of physical health on different types of mental health is also heterogeneous. To be specific, the effects of air pollution on depression and anxiety are partly mediated by physical health; whereas the effect on happiness is not. These findings contribute to the understanding of air pollution on public health, and have significant implication for social and public health policy makers.

## Introduction

Mental health has become an important global public health problem and a prominent social problem especially during the COVID-19 pandemic. According to the Public Health Agency of Canada, mental health is the capacity to feel, think, and act in ways that enhance human ability to enjoy life and deal with challenges (1). Mental health has significant impacts on daily life. Among mental disorders, depression and anxiety are two of the most common and psychological diseases, which affect people's physical and mental health leading to other disease and social consequences. The World Health Organization (WHO) reports that 92% of the world's population was living in an area where air quality exceeds WHO limits (2), which stimulates research on how air pollution affects mental health. In this paper, types of mental health refer to happiness, depression and anxiety disorder that are most commonly used by researchers (3–6). This research aims to examine the impact of air pollution on different types of mental health and the underlying economic and social factors and the mechanisms. The results will inform policy making for effective prevention and intervention mechanisms of mental health.

Mental disorders are conceptualized as behavioral or psychological syndromes that occur in a person in response to distress, disability or suffering, which include manic-depressive illness, schizophrenia, substance Abuse, post-traumatic stress and organically a disorder usually involving anxiety and depression (7). There are certain factors that cause mental disorders. On the one hand, some factors of individuals, family and social determinants, such as gender (8, 9), age (10), marital status (11), education level (12), physical status (13), unemployment (14, 15), incomes (16, 17), family status (18, 19), and so on.

On the other hand, many studies have explained the “Easterlin Paradox” from the perspectives of omitted variables. Some social factors, such as: economic status, occupation, social support, public welfare, especially for people's living environment (20, 21), are also found related to mental health. It is widely speculated that severe environmental pollution not only harms body functioning, but also negatively affects people's mental health (22–24). According to the extant studies, environmental pollution affects human nervous system with notable consequences on mental health. According to the extant studies, environmental pollution affects the human nervous system with remarkable consequences on their mental health, as well as impact on the generating a social stress related to unhealthy and poor living (24, 25), or act as a psychogenic agent since psychogenic disorders are the result of stress, shock, or any kind of psychological traumas in childhood, adolescence or adulthood (7). In general, these studies show that the influencing factors of mental health are varied, including family environment, social environment, and own conditions. However, these researches ignore air pollution or environmental pollution as an important factor.

Studies concerning the impact of environmental pollution on mental health mainly focus on air pollution leading to different mental disorders. Researchers have studied the effects of air pollution on life satisfaction, happiness, schizophrenia, autism, depression and negative emotions concerning older adults, adolescents and children. For instance, psychological research shows that air pollution can impair life satisfaction and subjective well-being (22, 26–32), which leads individuals to negative emotions (33). Gu et al. verified that the concentration of PM2.5 in the air leads to four types of negative emotions of tension, depression, weakness and restlessness (34). Mendoza et al. examined socio-economic data with annual mean PM10 and PM2.5 data, and found that air pollution significantly reduces life satisfaction among Chileans (35). A study conducted in Denmark e(36) found that pollution exposure with higher concentrations of oxy-nitrogen and NO2 in childhood were highly associated with the risk of schizophrenia (36). Earlier studies also suggested that concentrations of PM2.5 and PM10 were significantly correlated with autism spectrum disorders for children after birth within 3 years (37). Exposure to air pollution for a long time increases the probability of chronic mental diseases, and significantly increase their probability of mental disorders with physical health problems (38). These studies have reached a consistent conclusion that air pollution has a negative impact on different mental health and mental disease in different countries. However, it is also unclear whether there is difference in the impact of air pollution on different types of mental health.

In addition, some scholars also paid attention to the impact of subjective air pollution on mental health. Subjective air pollution refers to the subjective evaluation of the objective air quality, which has a direct impact on people's mental state (3, 4). For example, the concentrations pollutants of SO_2_, NO_2_, PM_2.5_, or PM_10_ in the same objective air environment may lead to different subjective air pollution indexes because different individuals may have different sensitivity to air pollution. Rehdanz and Maddison denoted that subjective air pollution and subjective noise pollution had a significant negative effect on German residents' well-being (6). Mackerron and Mourato proved that objective and subjective air pollution significantly reduced people's happiness by taking London residents' self-rated air pollution level of their street or community as a measure of subjective air pollution (5).

There are gaps in the literature on this contemporary topic. To note a few, studies analyzing the heterogeneous impact of air pollution on subjective well-being from perspectives of income levels, regional environmental laws and regulations, gender, and region (30, 39), inadequately consider different types of mental health. The impact of air pollution on mental health correlates with the subjective cognitive ability (40), which is directly related to the level of education, but there is no empirical study to analyze the impact of air pollution on mental health of groups with different education levels. There is little research on the influence of population density on the relationship between air pollution and mental health. It is well-known that air pollution affects physical health—e.g., air pollutants can reach the brain through the blood-brain barrier, or arrive their cerebrum- along the olfactory nerve, then trigger neuroinflammation when air pollution is severe (41). Besides, air pollution brought adverse effects such as man-made bad climate change (sulfur particles reflect sunlight and cause temperature drop) (42), as well as increased the incidence rate and premature death risk of cardiovascular and respiratory diseases (43, 44), or other immune function degradation (42, 45–48). These physical health problems further influence people's mental health. The mediating role of physical health in the effects of air pollution on different types of mental health has not been fully explored.

From the above, although many studies proved that air pollution has an impact on mental health, it still remains unclear how air pollution influences different types of mental health all over the world, and the underlying economic and social factors. For example, it is unclear whether there are differences in the impact of air pollution on different types of mental health under different income levels. In addition, people's different education level may lead to different subjective feeling and cognition for air pollution, which may cause different impact of air pollution on mental health. Moreover, population density may also contribute to mental health. Hence, this paper aims to address these issues, and the results will help bridge the gaps outlined above.

The remainder of this paper is arranged as follows: Section Research Methodology introduces research methods, variable selection, data sources, and basic tests. Section Results reports empirical results, which including the heterogeneous impacts of air pollution on different types of mental health, examined under different income level, and the heterogeneity of mediating effect. The subsequent section Discussion discusses the results with a comparison to the existing research. Lastly, section Conclusions concludes our paper with policy recommendations, as well as the shortcomings and further prospects.

## Research Methodology

### Empirical Strategy

#### Benchmark Regression Model

In this empirical study, the dependent variables are happiness, depression and anxiety. The data of happiness is obtained through the questionnaire, and its observation value is >0. The data of depression and anxiety are expressed as the increment of the prevalence of depression and anxiety, and the value ranges from−1 to 1. To a certain extent, the dependent variable is limited, that is, the data distribution of mental health is not continuous, but truncated. Besides, the Tobit model is a limited value dependent variable model, so it can be used to investigate the impact of air pollution on mental health (49–52). Because a consistent unbiased estimator cannot be obtained from the fixed effect Tobit model, the random effect Tobit model that can do better. The basic form of the panel Tobit model is constructed as below:


(1) $$\begin{array}{l} mental_{it}=\max\left(0,\beta_{0}+\beta_{1}*air_{it}+\lambda *Control_{it}\right) \end{array}$$

Where *mental* represents mental health, which including happiness, depression and anxiety; *air* stands for air pollution; *control* denotes for a series of control variables; β_0_ is the constant term, β_1_ and λ are the regression coefficients.

#### Verification Model of Mediating Effect

Air pollution is one of the causes to many diseases (53, 54). Physical health affects mental health. In order to further explore the heterogeneity of the mediating effect of physical health in the impact of air pollution on different types of mental health, with reference to Li et al. (55), the following mediating effect test models are constructed.


(2) $$\begin{array}{lll} mental_{it} & = & \max\left(0,\beta_{0}+\beta_{1}*air_{it}+\lambda *Control_{it}\right) \end{array}$$

(3) $$\begin{array}{lll} physical_{it} & = & \max\left(0,\beta_{0}+\beta_{2}*air_{it}+\lambda *Control_{it}\right) \end{array}$$

(4) $$\begin{array}{l} mental_{it}=\max(0,\beta_{0}+\beta_{3}*air_{it}+\beta_{4}*physical_{it}\\ \;+\lambda *Control_{it}) \end{array}$$

Where formula (2), (3), and (4) are the models of mediating effect. Among them, *physical* represents physical health, which is selected as proxy variable from life expectancy. Based on these formulas, the improved causal test and stepwise regression method were used based on the work of Wen and Ye (56). The specific inspection steps are as follows:

Step 1 is to test the regression coefficient β_1_ in formula (2). If β_1_ is significant, it continues step 2, otherwise, the test will be stopped.

Step 2 is to test regression coefficient β_2_ and β_4_, respectively in formula (3) and (4). If β_2_ and β_4_ are significant, it has a mediating effect and continues to step 3. Otherwise, the test will be stopped for lacking a mediating effect.

Step 3 is to test regression coefficient β_3_ in formula (4). If β_3_ is significant, it means physical health has partial mediating effect, and there may be other mediating effects on the impact of air pollution on mental health; If it is not significant, it indicates that there is a complete mediating effect on physical health, that is, air pollution does not directly affect mental health, but completely affects mental health through physical health.

### Data

#### Variables Selection and Data Resource

(1) Explained variable. The explained variable in this paper is mental health, including happiness, depression and anxiety. Proxy variables for happiness are measured in the Gallup World Poll (https://www.gallup.com/topic/world_poll.aspx). The data refers to the national average of responses to life evaluation questions. The prevalence of depression and anxiety disorders are determined by the increase in the proportion of the sample with depressive and anxiety disorders divided by the total number; namely, the increase in the prevalence of depression and anxiety disorders (57). They come from Global Burden of Disease (GBD) (https://vizhub.healthdata.org/gbd-compare/).
(2) Explanatory variables. The association between air pollution and mental health has been well-documented in the medical literature, which showed the correlation between long-term exposure to fine particles (mainly related to road traffic) and mental health (58–61). At the same time, particulate matter has become a major problem of air pollution. Compared with other pollutants, PM2.5 has a greater impact on people, and is more harmful to human health. The main components of particulate matter are sulfate, nitrate, ammonia, sodium chloride, carbon, mineral dust and water, which are composed of a complex mixture of solid and liquid particles of organic and inorganic substances suspended in the air. Most of the urban and rural populations in both developed and developing countries are currently exposed to particulate matter at high enough levels to have an impact on mental health. Therefore, the average annual exposure concentration of PM_2.5_ is selected as the air pollution measure in this paper. This indicator is based on data drawn from the World Bank (WD) database. The database is updated and covers continuous data for nearly 200 countries from 2010 to 2017 on the annual average exposure levels of fine particulate matter PM_2.5_ across the world. The World Health Organization (WHO) issued Air Quality Guidelines in 2005 with an effort to keep the concentration as low as possible, and set a guideline value for particulate matter PM_2.5_ for the first time (http://www.euro.who.int/en/health-topics/environment-and-health/air-quality/publications/pre2009/air-quality-guidelines). Specifically, the standard value of annual average concentration is 10 μg/ m^3^; that is to say, when the concentration is lower than this value, the harm to health can be minimized. Based on this, the average annual exposure concentration of PM_2.5_ that is higher than 10 g/m^3^ in countries are selected as sample countries according to this guideline values.
(3) Control variables. According to relevant literature (62, 63), the control variables in this paper include income level, social support, unemployment, generosity, positive emotion and negative emotion. Among them, income level is measured by per capita values for gross national income (64), and the data comes from the World Bank database; Social support, generosity, positive and negative emotions are from the Gallup World Poll (65); Unemployment data is from the World Development Indicators Database (WDI).
(4) Other variables. This paper selects subjective and objective factors as grouping variables. The level of education is a subjective factor, which is measured by the Mean years of schooling (year) from the United Nations Development Programme; the population density is an objective factor, which equals the total population of each country divided by the administrative area; that is, the population per 10,000 km. In addition, the mediating effect of physical health is further explored. The proxy variable of physical health is life expectancy, which comes from the World Health Organization (WHO). The measurements and sources of all variables are shown in Table 1 as below.

**Table 1:** Variables measurement and data source.

**Type of variable**	**Variable**	**Measurement**	**Range**	**Data source**
Explained variable	Happiness	Imagine a ladder, with steps numbered from 0 at the bottom to 10 at the top. The top of the ladder represents the best possible life for you and the bottom of the ladder represents the worst possible life for you. On which step of the ladder would you say you personally feel you stand at this time.	[0, 10]	GWP
	Depression	Rate of increase in prevalence of depression disorders	[−1, 1]	GBD
	Anxiety	Rate of increase in prevalence of anxiety disorders	[−1, 1]	GBD
Explanatory variables	Air pollution	PM2.5 Annual exposure	-	WB
Control variables	Income	Per capita values for gross national income	-	WD
	Social support	National average of the binary responses to the GWP question “If you were in trouble, do you have relatives or friends you can count on to help you whenever you need them, or not?”	[0, 1]	GWP
	Unemployment	Unemployment, total (% of total labor force)	[0, 1]	WDI
	Generosity	Residual of regressing national average of response to the GWP question “Have you donated money to a charity in the past month?” on GDP per capita.	-	GWP
	Negative affect	Average measures of previous-day affect for worry, sadness, and anger for all waves.	[0, 1]	GWP
	Positive affect	Average of three positive affect measures for happiness, laughter and enjoyment in the Gallup World Poll.	[0, 1]	GWP
Grouping variable	Education	Mean years of schooling (year)	-	UNDP
	population density	Total population/administrative area	-	WDI
Mediate variable	Physical health	Healthy life expectancies at birth	-	WHO

*GWP, Gallup World Poll; GBD, Global Health Data; WDI, World Development Indicators; WB, World Bank; UNDP, United Nations Development Programme; WHO, World Health Organization*.

#### Sample and Data Description Statistics

The panel data includes 51 countries from 2010 to 2017, which is based on the following reasons. Firstly, due to the limitation of PM_2.5_ availability, the annual data of sample interval is from 2010 to 2017. Secondly, with the continuity of the survey data, the indicators to be used are the indicators with continuity since 2010; however, some countries miss some of indicators. Thirdly, in order to make the sample more representative, this paper selects the sample countries whose concentration of PM_2.5_ is higher than the standard value (although the minimal risk to humans is lower than the standard value). Based on data availability of time dimension and data integrity of sample countries, 51 countries are finally selected as the research target. In order to eliminate the influence of heteroscedasticity, the logarithmic processing is carried out on partial data, including air pollution, per capita national income level and population density. The descriptive statistics of all data based on the full sample are shown in Table 2.

**Table 2:** Descriptive statistics of full sample.

**Variable**	**Obs**	**Mean**	**Std. Dev**.	**Min**	**Max**
Happiness	408	5.546	1.014	2.903	7.788
Deppression	408	0.000118	0.000157	−0.0007	0.0005
Anxiety	408	4.099E−05	0.000123	−0.0006	0.0003
Air pollution	408	3.25	0.629	2.227	5.317
Income	408	8.417	1.268	5.826	11.069
Unemployment	408	0.061	0.043	0.003	0.271
Social support	408	0.823	0.098	0.510	0.975
Generosity	408	−0.015	0.148	−0.303	0.549
Positive effect	408	0.737	0.103	0.450	0.944
Negative effect	408	0.264	0.078	0.112	0.483
Education	408	8.409	2.904	1.4	14.1
Population density	408	4.331	1.202	1.221	7.112
Physical health	408	0.638	0.059	0.483	0.749

#### Stationary Test

The stationary tests on all index data are conducted using Levin-Lin-chu unit-root test and Fisher ADF unit-root test before the regression model. This is to avoid the emergence of false regression problem. The priory test results are shown in Table 3.

**Table 3:** Results of the panel unit root test.

**Variables**	**LLC test**	**Fisher-ADF test**
Happiness	−18.497^***^ (0.000)	262.868^***^ (0.000)
Depression	−53.126^***^ (0.000)	173.128^***^ (0.000)
Anxiety	−44.688^***^	238.799^***^ (0.000)
Air pollution	−9.448^***^ (0.000)	194.574^***^ (0.000)
Income	−13.975^***^ (0.000)	255.679^***^ (0.000)
Unemployment	−17.672^***^ (0.000)	222.382^***^ (0.000)
Social support	−22.217^***^ (0.000)	260.392^***^ (0.000)
Generosity	−24.884^***^ (0.000)	228.32^***^ (0.000)
Positive effect	−18.372^***^ (0.000)	266.964^***^ (0.000)
Negative effect	−16.279^***^ (0.000)	247.585^***^ (0.000)

*This table summarizes panel unit-root tests for all related variables in 51 countries.*

*LLC, Levin-Lin-Chu unit-root test; Fisher-ADF, fisher ADF unit-root tests.*

^***, **, *^Indicate significant at the 1, 5, and 10% levels, respectively.

*Standard errors are in parentheses. The sample period is from 2010 to 2017*.

Table 3 reports the unit root test results of happiness, depression, anxiety, air pollution, unemployment, social support, generosity, positive effect, and negative effect. Two test results for the null hypothesis contains unit roots. The first is the Levin-Lin-Chu unit root test, and the second is the Fisher augmented Dickey Fuller *t*-test. The criterion is: if the unit root test confirms that, it is 0 at the 5% significance level; thus, the variable is stationary. In both cases, the null hypothesis is rejected at the 1% significance level. Therefore, we can conclude that all variables are stationary.

## Results

### The Heterogeneous Impact of Air Pollution on Different Types of Mental Health

The panel Tobit model (1) is used to empirically test the selected samples. Firstly, the maximum likelihood estimate (MLE) method is used to estimate the parameters of the Tobit random effect model by selecting happiness, depression and anxiety as explained variables. In order to test the robustness, control variables are added gradually to estimate the model for exploring the interference factors. The control variables are unemployment, social support, generosity, positive affect, negative affect, and income level. The regression results are shown in the following Tables 4–**6**.

**Table 4:** Regression results of air pollution on happiness under complete sample.

	**(1)**	**(2)**	**(3)**	**(4)**	**(5)**	**(6)**	**(7)**
	**Happiness**	**Happiness**	**Happiness**	**Happiness**	**Happiness**	**Happiness**	**Happiness**
Air	−0.413^***^	−0.394^***^	−0.335^***^	−0.345^***^	−0.342^***^	−0.344^***^	**−0.128**
	(0.115)	(0.115)	(0.109)	(0.108)	(0.107)	(0.109)	(0.097)
Unemp		−4.999^***^	−5.301^***^	−5.016^***^	−4.718^***^	−4.725^***^	−4.838^***^
		(1.423)	(1.376)	(1.377)	(1.385)	(1.386)	(1.183)
Soc			1.884^***^	1.906^***^	1.892^***^	1.907^***^	1.292^***^
			(0.422)	(0.419)	(0.418)	(0.437)	(0.424)
Gene				0.533^**^	0.517^**^	0.516^**^	0.559^**^
				(0.228)	(0.228)	(0.229)	(0.218)
Posi					0.596	0.601	0.721^*^
					(0.408)	(0.411)	(0.384)
Nega						0.0508	−0.293
						(0.425)	(0.411)
Income							0.521^***^
							**(0.0643)**
_cons	6.886^***^	7.128^***^	5.406^***^	5.410^***^	4.954^***^	4.932^***^	0.363
	(0.391)	(0.396)	(0.537)	(0.533)	(0.616)	(0.643)	(0.822)
sigma_u	0.814^***^	0.833^***^	0.766^***^	0.764^***^	0.747^***^	0.746^***^	0.492^***^
	(0.0889)	(0.0923)	(0.0843)	(0.0838)	(0.0823)	(0.0829)	(0.0523)
sigma_e	0.320^***^	0.313^***^	0.308^***^	0.306^***^	0.306^***^	0.306^***^	0.303^***^
	(0.0121)	(0.0119)	(0.0117)	(0.0116)	(0.0116)	(0.0116)	(0.0114)
*N*	408	408	408	408	408	408	408
LR test (*p*-value)	0.000	0.000	0.000	0.000	0.000	0.000	0.000

*happ, dep, anx, unemp, soc, gene, posi, nega represent happiness, depression, anxiety, unemployment, social support, generosity, positive effect, and negative effect, respectively. This is a regression with panel Tobit model for a balanced panel explaining the relationship between air pollution and happiness from 2010 to 2017.*

^***, **, *^Indicate significant at the 1, 5, and 10% levels, respectively.

*Z or T-value is in parentheses. The sample period is from 2010 to 2017*.

Table 4 shows that the significance of the estimated value of regression coefficient of air pollution on happiness has changed from significant to insignificant in the process of gradually adding control variables under complete sample. Specifically, the regression coefficients of air pollution on happiness are significantly negative when control variables such as unemployment, social support, generosity, positive emotion, and negative emotion are gradually added; whereas, it is not significant after adding control variable of income level. Therefore, income level may influence the impact of air pollution on happiness through this empirical research. In other words, the significant impact of air pollution on happiness is related to income level.

Next, Table 5 reports that the estimated value of regression coefficient of air pollution on depression is persistently significantly positive when a series of control variables such as unemployment, social support, generosity, positive affect, negative affect, and income level, are gradually added. This shows that air pollution significantly promotes the increase of depression.

**Table 5:** Regression results of air pollution on depression under complete sample.

	**(1)**	**(2)**	**(3)**	**(4)**	**(5)**	**(6)**	**(7)**
	**Depression**	**Depression**	**Depression**	**Depression**	**Depression**	**Depression**	**Depression**
Air	0.0000594^***^	0.0000593^***^	0.0000435^*^	0.0000439^**^	0.0000447^**^	0.0000393^*^	0.0000481^**^
	(0.0000215)	(0.0000215)	(0.0000223)	(0.0000223)	(0.0000223)	(0.0000224)	(0.0000245)
Unemp		−0.0000614	−0.00000143	−0.0000254	−0.00000899	−0.0000592	−0.0000829
		(0.000296)	(0.000294)	(0.000296)	(0.000297)	(0.000299)	(0.000300)
Soc			−0.000249^**^	−0.000250^**^	−0.000253^**^	−0.000185^*^	−0.000210^**^
			(0.0000984)	(0.0000984)	(0.0000986)	(0.000102)	(0.000105)
Gene				−0.0000343	−0.0000358	−0.0000411	−0.0000390
				(0.0000540)	(0.0000541)	(0.0000538)	(0.0000538)
Posi					0.0000431	0.0000546	0.0000534
					(0.0000969)	(0.0000961)	(0.0000960)
Nega						0.000248^**^	0.000231^**^
						(0.0001000)	(0.000102)
Income							0.0000148
							(0.0000167)
_cons	−0.0000674	−0.0000633	0.000190	0.000190	0.000157	0.0000474	−0.0000783
	(0.0000725)	(0.0000751)	(0.000125)	(0.000125)	(0.000145)	(0.000151)	(0.000207)
sigma_u	0.000133^***^	0.000132^***^	0.000130^***^	0.000130^***^	0.000129^***^	0.000133^***^	0.000131^***^
	(0.0000137)	(0.0000137)	(0.0000135)	(0.0000136)	(0.0000138)	(0.0000142)	(0.0000141)
sigma_e	0.0000755^***^	0.0000755^***^	0.0000751^***^	0.0000750^***^	0.0000751^***^	0.0000742^***^	0.0000742^***^
	(0.00000283)	(0.00000283)	(0.00000281)	(0.00000281)	(0.00000282)	(0.00000279)	(0.00000279)
*N*	408	408	408	408	408	408	408
LR test (*p*-value)	0.000	0.000	0.000	0.000	0.000	0.000	0.000

*happ, dep, anx, unemp, soc, gene, posi, nega represent happiness, depression, anxiety, unemployment, social support, generosity, positive effect, and negative effect, respectively. This is a regression with panel Tobit model for a balanced panel explaining the relationship between air pollution and happiness from 2010 to 2017.*

^***, **, *^Indicate significant at the 1, 5, and 10% levels, respectively.

*Z or T–value is in parentheses. The sample period is from 2010 to 2017*.

Moreover, the results in Table 6 are similar to Table 4. Under full sample, the significance of the estimated value of regression coefficient of air pollution on anxiety changed from significant to insignificant when it adds control variables gradually. Specifically, the regression coefficient of air pollution on anxiety is significantly positive when control variables such as unemployment, social support, generosity, positive emotion, and negative emotion are added; however, it becomes insignificant after adding one more control variables of income level. From the above, the empirical results show that the impact of air pollution on anxiety is related to income level.

**Table 6:** Regression results of air pollution on anxiety under complete sample.

	**(1)**	**(2)**	**(3)**	**(4)**	**(5)**	**(6)**	**(7)**
	**Anxiety**	**Anxiety**	**Anxiety**	**Anxiety**	**Anxiety**	**Anxiety**	**Anxiety**
Air	0.0000465^***^	0.0000449^***^	0.0000370^**^	0.0000364^**^	0.0000388^**^	0.0000402^***^	**0.0000206**
	(0.0000159)	(0.0000152)	(0.0000155)	(0.0000154)	(0.0000155)	(0.0000155)	(0.0000165)
Unemp		−0.00117^***^	−0.00113^***^	−0.00106^***^	−0.00102^***^	−0.00101^***^	−0.000921^***^
		(0.000207)	(0.000207)	(0.000208)	(0.000209)	(0.000210)	(0.000209)
Soc			−0.000129^*^	−0.000128^*^	−0.000134^*^	−0.000151^**^	−0.0000980
			(0.0000691)	(0.0000687)	(0.0000688)	(0.0000711)	(0.0000727)
Gene				0.0000850^**^	0.0000819^**^	0.0000832^**^	0.0000796^**^
				(0.0000376)	(0.0000376)	(0.0000376)	(0.0000372)
Posi					0.0000823	0.0000771	0.0000855
					(0.0000653)	(0.0000655)	(0.0000645)
Nega						−0.0000658	−0.0000288
						(0.0000693)	(0.0000695)
Income							−0.0000314^***^
							(0.0000107)
_cons	−0.0000999^*^	−0.0000236	0.000106	0.000104	0.0000378	0.0000680	0.000331^**^
	(0.0000535)	(0.0000532)	(0.0000869)	(0.0000864)	(0.000101)	(0.000106)	(0.000139)
sigma_u	0.0000945^***^	0.0000900^***^	0.0000882^***^	0.0000865^***^	0.0000856^***^	0.0000859^***^	0.0000806^***^
	(0.0000100)	(0.00000961)	(0.00000940)	(0.00000933)	(0.00000930)	(0.00000931)	(0.00000880)
sigma_e	0.0000549^***^	0.0000529^***^	0.0000528^***^	0.0000525^***^	0.0000525^***^	0.0000524^***^	0.0000523^***^
	(0.00000206)	(0.00000199)	(0.00000198)	(0.00000198)	(0.00000198)	(0.00000197)	(0.00000197)
*N*	408	408	408	408	408	408	408
LR test (*p*-value)	0.000	0.000	0.000	0.000	0.000	0.000	0.000

*happ, dep, anx, unemp, soc, gene, posi, nega represent happiness, depression, anxiety, unemployment, social support, generosity, positive effect, and negative effect, respectively. This is a regression with panel Tobit model for a balanced panel explaining the relationship between air pollution and happiness from 2010 to 2017.*

^***, **, *^Indicate significant at the 1, 5, and 10% levels, respectively.

*Z or T–value is in parentheses. The sample period is from 2010 to 2017*.

In addition, the ordinary panel fixed effect model and ordinary panel random effect model are also used to test their robustness. The results of the robustness test in Table 7 show that the estimation results of common panel fixed effect and random effect are consistent with the panel Tobit model, which means air pollution can increase the degree of depression. However, the impact of air pollution on happiness and anxiety is not significant when it takes income level into consideration. From the above, it proves that the effects of air pollution on different types of mental health are heterogeneous. Specifically, air pollution has a significant promoting role in depression; whereas the significant impact of air pollution on happiness and anxiety is related with income level.

**Table 7:** Robustness test result of replacing estimation method.

	**(1)**	**(2)**	**(3)**
	**Happiness**	**Depression**	**Anxiety**
Air	−0.0175	0.0000625^**^	0.0000304
	(0.130)	(0.0000301)	(0.0000216)
Control variables	Yes	Yes	Yes
Constant	Yes	Yes	Yes
Time effect	Yes	Yes	Yes
Individual effect	Yes	Yes	Yes
*N*	408	408	408
*R* ^2^	0.128	0.181	0.185

*This is a regression with ordinary panel regression model for a balanced panel explaining the relationship between air pollution and mental health from 2010 to 2017.*

^***, **, *^Indicate significant at the 1, 5, and 10% levels, respectively.

*Z or T-value is in parentheses. The sample period is from 2010 to 2017*.

### The Heterogeneous Impact of Air Pollution on Mental Health Under Different Income Level

#### Data Grouping and Descriptive Statistics

Based on the above, the heterogeneity of the impact of air pollution on happiness under different income levels is further analyzed. According to the classification standard of the World Bank, the sample countries are divided into 29 high-income countries and 22 low-income countries. Specifically, based on its grouping standards, the high-income and upper middle-income countries are classified into the high-income group, and the low-income and lower middle-income countries are classified into the low-income group. The descriptive statistics data after grouping are shown in Table 8.

**Table 8:** Descriptive statistics data of different income levels grouping.

**Group**	**Variable**	**Obs**	**Mean**	**Std. Dev**.	**Min**	**Max**
High–income	Happiness	232	6.117	0.795	3.661	7.788
group	Depression	232	0.00012	0.00017	−0.0007	0.0005
	Anxiety	232	6.41E−06	0.00011	−0.0006	0.0003
	Air pollution	232	2.925	0.396	2.227	4.067
	Income	232	9.33	0.812	7.92	11.069
	Social support	232	0.873	0.062	0.66	0.975
	Generosity	232	−0.037	0.164	−0.303	0.549
	Positive	232	0.76	0.106	0.45	0.944
	Negative	232	0.254	0.075	0.112	0.464
	Education	232	9.932	2.174	4.6	14.1
	Density	232	4.233	1.143	1.799	6.269
	Physical health	232	0.673	0.042	0.505	0.749
Low–income	Happiness	176	4.793	0.745	2.903	6.74
group	Depression	176	0.00014	0.000135	−0.00035	0.00044
	Anxiety	176	0.00011	0.000095	−0.00009	0.00033
	Air pollution	176	3.678	0.623	2.711	5.317
	Income	176	7.213	0.553	5.826	8.152
	Social support	176	0.757	0.097	0.51	0.914
	Generosity	176	0.016	0.117	−0.206	0.418
	Positive	176	0.708	0.091	0.513	0.876
	Negative	176	0.277	0.081	0.123	0.483
	Education	176	6.401	2.498	1.4	11.6
	Density	176	4.459	1.266	1.221	7.112
	Physical health	176	0.592	0.046	0.483	0.672

Table 8 demonstrates that the four variables of air pollution, happiness, the increment of depression and anxiety prevalence have heterogeneity under the groups of high-income and low-income. Specifically, in the high-income group, the average value of air pollution is 2.925, happiness is 6.117, the increment prevalence of depression is 0.00012, and anxiety is 6.41e-06, respectively; however, in the low-income group, the average value of air pollution is 3.678, happiness is 4.793, the increment prevalence of depression is 0.00014, and anxiety is 0.00011, respectively. Based on the above results, when compared with the high-income group, the low-income group has a higher level of air pollution, depression, and anxiety; whereas it has lower happiness.

**Figure 1:**
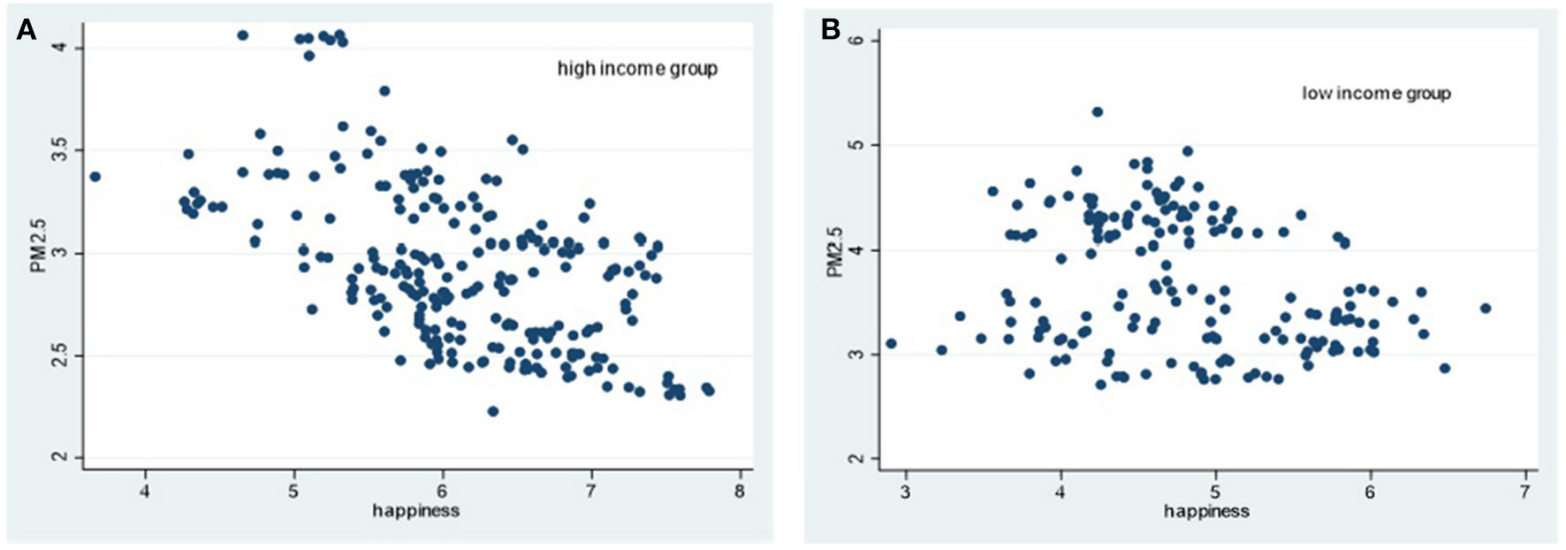
Scatter diagram of relationship between PM2.5 concentration and happiness in different income groups. **(A)** High income group. **(B)** Low income group.

**Figure 2:**
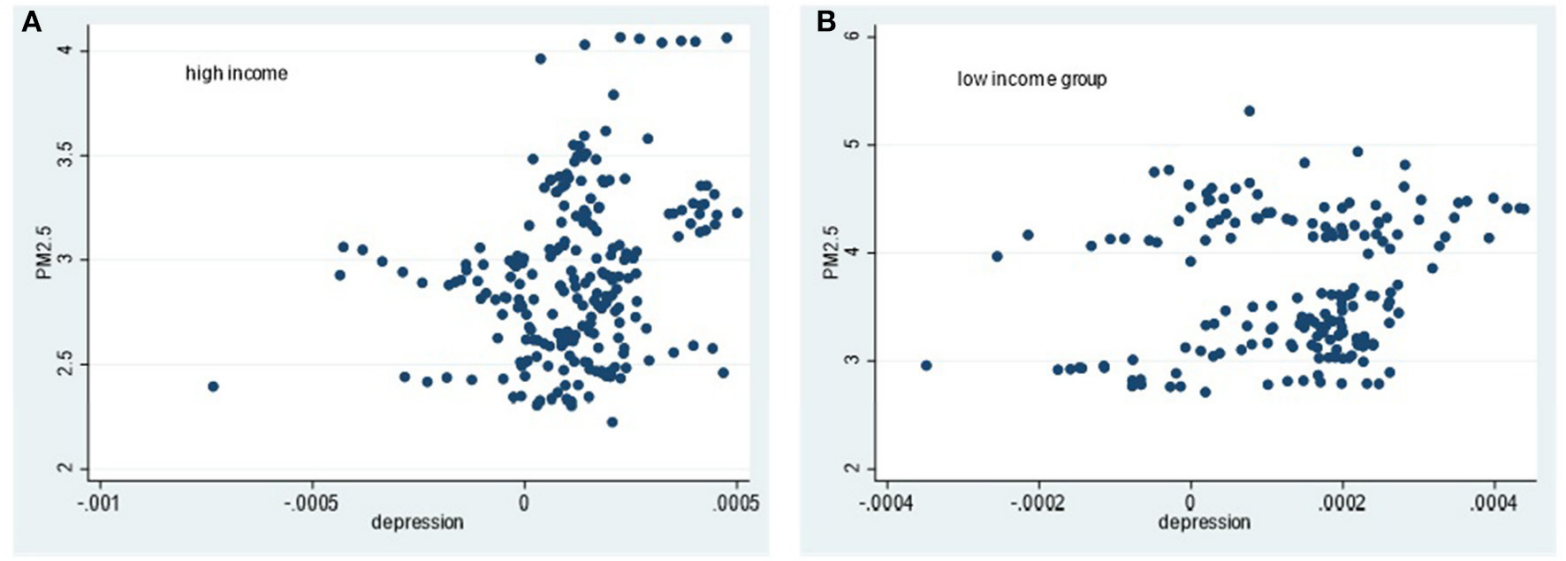
Scatter diagram of relationship between PM2.5 concentration and depression in different income groups. **(A)** High income group. **(B)** Low income group.

**Figure 3:**
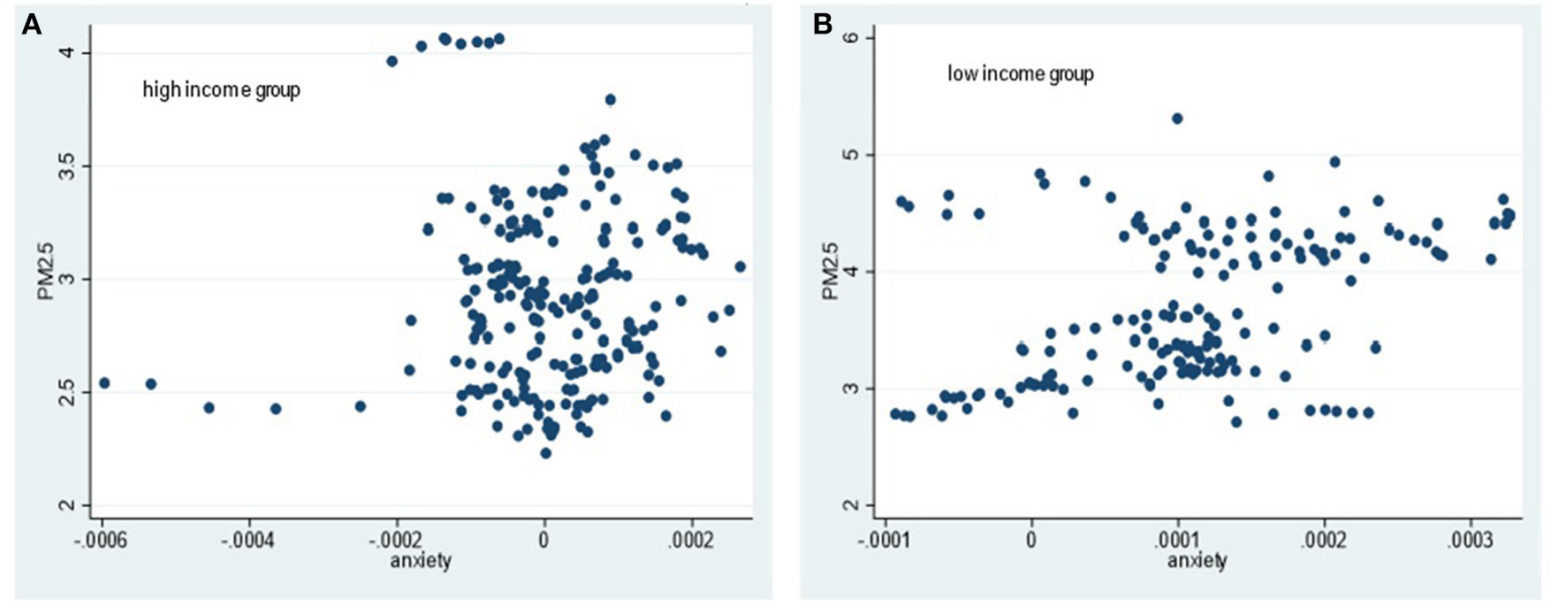
Scatter diagram of relationship between PM2.5 concentration and anxiety in different income groups. **(A)** High income group. **(B)** Low income group.

Simultaneously, the heterogeneous impact of air pollution on different types of mental health under different income levels is further analyzed by drawing scatter diagrams for the explained variables and explanatory variables. The explained variables are happiness, depression and anxiety. The core explanatory variable is air quality, which is measured by PM_2.5_ annual concentration. The correlation judgments are according to Figures 1–3 as below.

From Figures 1–3, in high-income countries, PM_2.5_ concentration is negatively related with the scatter of happiness; whereas it has no obvious relations with depression and anxiety. In low-income countries, there is no obvious rule between PM_2.5_ concentration and happiness, but a positive correlation between PM_2.5_ concentration and depression and anxiety, respectively.

#### Results of Heterogeneity Test of Income Level

By taking per capita national income as an index to measure income differences, the heterogeneous impact of air pollution on different types of mental health that are caused by income differences is examined. Tobit model (1) of benchmark panel by the MLE method is estimated in Table 9 as below.

**Table 9:** Regression results of air pollution on mental health with different income levels.

	**(1)**	**(2)**	**(3)**	**(4)**	**(5)**	**(6)**
	**High–income** **sample group**	**Low-income** **sample group**	**High-income** **sample group**	**Low-income** **sample group**	**High-income** **sample group**	**Low-income** **sample group**
	**Happiness**	**Happiness**	**Depression**	**Depression**	**Anxiety**	**Anxiety**
Air	−0.439^***^	0.0222	0.000044	0.000047^*^	−0.000022	0.000032^**^
	(0.145)	(0.132)	(0.00005)	(0.00003)	(0.00004)	(0.00001)
Control variables	Yes	Yes	Yes	Yes	Yes	Yes
Constant	Yes	Yes	Yes	Yes	Yes	Yes
sigma_u	0.449^***^	0.466^***^	0.00016^***^	0.000088^***^	0.000086^***^	0.000064^***^
	(0.0627)	(0.0770)	(0.000023)	(0.000015)	(0.000013)	(0.000011)
sigma_e	0.226^***^	0.374^***^	0.000074^***^	0.000071^***^	0.000063^***^	0.000031^***^
	(0.0113)	(0.0214)	(0.0000037)	(0.0000041)	(0.0000031)	(0.0000018)
*N*	232	176	232	176	232	176
LR test (*p*-value)	0.000	0.000	0.000	0.000	0.000	0.000

^***, **, *^Indicate significant at the 1, 5, and 10% levels, respectively.

*Z or T-value is in parentheses. The sample period is from 2010 to 2017*.

Table 9 shows the results of the benchmark regression model under the high-income and low-income sample groups. Column (1) to (6) list the results of the impact of air pollution on happiness, depression and anxiety. The regression coefficient of air pollution on happiness is −0.439, which is significant at the 1% significance level; whereas the regression coefficients of air pollution on depression and anxiety are not significant in the sample of high-income groups. However, in low-income countries, the regression coefficients of air pollution on anxiety and depression are 0.000047 and 0.000032, which are significant at the significance level of 10 and 5%, respectively; whereas the regression coefficient of air pollution on happiness is not significant. These results show that the impact of air pollution on mental health is heterogeneous under different income levels. Specifically, the impact of air pollution on happiness only has a significant negative impact in high-income countries; whereas the effects of air pollution on depression and anxiety are significantly positive only in low-income countries.

### Heterogeneity Test of Different Levels of Education and Population Density Under Different Income Levels

In order to fully consider the heterogeneous impact of air pollution on mental health under other subjective and objective factors of different income levels, the following analysis is carried out. First, education level and population density are selected as two variables. Then, samples are divided into two income level groups with significant effects of air pollution on various types of mental health based on section The Heterogeneous Impact of Air Pollution on Different Types of Mental Health. The values of education level (or population density) is in order from the smallest to largest for each income level group. Next, the groups with different income levels are further divided into upper and lower quantile groups by 50% quantile. The upper ones are the groups with high education level (or high population density); and the lower ones are the groups with low level education (or low population density).

From the above, the impact of air pollution on happiness is significant only in the high-income group, whereas its impact on depression and anxiety are significant only in the low-income group. Based on this, a further investigation reveals that the effects of air pollution on depression and anxiety are also verified in low-income countries regardless of education groups. Finally, in view of model (1), the estimating results of the panel Tobit random effect model by the MLE estimation method are shown in Tables 10, 11.

**Table 10:** Heterogeneity test of education level under different incomes.

	**(1)**	**(2)**	**(3)**	**(4)**	**(5)**	**(6)**
	**High-income and** **high-education** **sample group**	**High-income and** **low-education** **sample group**	**Low-income and** **high-education** **sample group**	**Low-income and** **low-education** **sample group**	**Low-income and** **high-education** **sample group**	**Low-income and** **low-education** **sample group**
	**Happiness**	**Happiness**	**Depression**	**Depression**	**Anxiety**	**Anxiety**
Air	−0.569^***^	−0.275	−0.000044	0.000032	0.000055	0.000022
	(0.215)	(0.205)	(0.000090)	(0.000027)	(0.000041)	(0.000014)
Control variables	Yes	Yes	Yes	Yes	Yes	Yes
Constant	Yes	Yes	Yes	Yes	Yes	Yes
sigma_u	0.396^***^	0.569^***^	0.0006^***^	0.000073^***^	0.000037^**^	0.000066^***^
	(0.0699)	(0.124)	(0.000056)	(0.000016)	(0.0000169)	(0.000012)
sigma_e	0.223^***^	0.208^***^	0.000041^***^	0.000075^***^	0.000019^***^	0.000032^***^
	(0.0142)	(0.0178)	(0.0000054)	(0.0000049)	(0.0000026)	(0.0000021)
*N*	149	83	35	141	35	141
LR test (*p*–value)	0.000	0.000	0.000	0.000	0.000	0.000

^***, **, *^Indicate significant at the 1, 5, and 10% levels, respectively.

*Z or T-value is in parentheses. The sample period is from 2010 to 2017*.

**Table 11:** Heterogeneity test of population density with different incomes.

	**(1)**	**(2)**	**(3)**	**(4)**	**(5)**	**(6)**
	**High-income and** **high-density** **sample group**	**High-income and** **low-density** **sample group**	**Low-income and** **high-density** **sample group**	**Low-income and** **low-density** **sample group**	**Low-income and** **high-density** **sample group**	**Low-income and** **low-density** **sample group**
	**Happiness**	**Happiness**	**Depression**	**Depression**	**Anxiety**	**Anxiety**
Air	−0.685^***^	−0.323	0.000052	0.000051^**^	0.000059^***^	0.000030^*^
	(0.185)	(0.221)	(0.000042)	(0.000026)	(0.000019)	(0.000017)
Control variables	Yes	Yes	Yes	Yes	Yes	Yes
Constant	Yes	Yes	Yes	Yes	Yes	Yes
sigma_u	0.570^***^	0.302^***^	0.000099^***^	0.00011^***^	0.000044^***^	0.000063^***^
	(0.121)	(0.0717)	(0.000024)	(0.000027)	(0.000011)	(0.000014)
sigma_e	0.185^***^	0.245^***^	0.000078^***^	0.000043^***^	0.000029^***^	0.000029^***^
	(0.0134)	(0.0174)	(0.0000062)	(0.0000039)	(0.0000023)	(0.0000026)
*N*	112	120	100	76	100	76
LR test (*p*-value)	0.000	0.000	0.000	0.000	0.000	0.000

^***, **, *^Indicate significant at the 1, 5, and 10% levels, respectively.

*Z or T-value is in parentheses. The sample period is from 2010 to 2017*.

Table 10 lists heterogeneity test results of education level under different incomes from Column (1) to Column (6). It shows that the estimated value of regression coefficient of air pollution on happiness is −0.569 in the sample of high income and high education level, which is significant at the 1% significance level; whereas the impact of air pollution on happiness is not significant under high income and low-level education sample. These manifest that in high-income countries, air pollution has a significant inhibitory effect on happiness in high-level educated samples; whereas it is not for the low educated one. In addition, in low-income countries, air pollution has no significant impact on depression and anxiety under different education levels; in other words, education level does not affect the impact of air pollution on depression and anxiety, which further indicates that income or economy may be the main causes for depression and anxiety.

Table 11 shows heterogeneity test of population density level under different incomes. The result shows that the estimated value of regression coefficient of air pollution on happiness is −0.685 in the samples of high income and high population density, which is significant at the significance level of 1%. However, the impact is not significant under the condition of high income and low population density. These results indicate that air pollution has a significant inhibitory effect on happiness in high-income countries with high population density, whereas it is not for countries with low population density.

In samples with low income and low population density, the estimated value of regression coefficient of air pollution on depression is 0.000051, which is significant at the significance level of 5%; that is, air pollution has a significant aggravating effect on depression under the condition of low income and low population density. However, under the condition of low income and high population density, the effect of air pollution on depression is not significant.

Furthermore, in low-income countries, the estimated value of regression coefficients of air pollution on anxiety in high and low population density countries are 0.000059 and 0.000030, respectively, which are significant at 1 and 10% significance levels. This manifests that in low-income countries, air pollution has a significant positive impact on anxiety regardless of population density; however, the impact on high-density countries is greater than the lower ones.

### Heterogeneous Mediating Effect Tests

The heterogeneity of the mediating effects of physical health on the effect of air pollution on happiness, depression and anxiety is tested by taking three strict steps mentioned in section Research Methodology. The test uses MLE mediating effect model. First, based on the significant impact of air pollution on happiness only in high-income countries, hence only the sample of high-income countries is selected to test the intermediate effect of physical health. To test the intermediate effect of physical health on the effect of air pollution on depression and anxiety, the sample of low income countries is selected as early results show a significant influence on depression and anxiety in low-income countries. Then, by taking happiness, depression and anxiety as explanatory variables, and physical health as mediating variables, the results of the Tobit model (2), (3), and (4) are estimated, respectively in Table 12.

**Table 12:** Test of mediating effect of physical health on the effect of air pollution on mental health.

	**(1)**	**(2)**	**(3)**	**(4)**	**(5)**	**(6)**	**(7)**	**(8)**	**(9)**
	**Happiness**	**Physical**	**Happiness**	**Depression**	**Physical**	**Depression**	**Anxiety**	**Physical**	**Anxiety**
Air	−0.439^***^	**−0.000739**	−0.441^***^	0.0000469^*^	**0.00104**	0.0000590^**^	0.0000317^**^	**0.00104**	0.0000312^**^
	(0.145)	(0.00428)	(0.145)	(0.0000250)	(0.00251)	(0.0000251)	(0.0000131)	(0.00251)	(0.0000133)
Physical			**0.435**			0.00172^***^			0.00112^***^
			(2.087)			(0.000573)			(0.000348)
Control variables	Yes	Yes	Yes	Yes	Yes	Yes	Yes	Yes	Yes
Constant	Yes	Yes	Yes	Yes	Yes	Yes	Yes	Yes	Yes
sigma_u	0.449^***^	0.0312^***^	0.446^***^	0.000088^***^	0.0407^***^	0.000093^***^	0.000064^***^	0.0407^***^	0.000072^***^
	(0.0627)	(0.00468)	(0.0632)	(0.000015)	(0.0063)	(0.000018)	(0.000011)	(0.00632)	(0.000013)
sigma_e	0.226^***^	0.00568^***^	0.226^***^	0.000071^***^	0.00515^***^	0.000068^***^	0.000031^***^	0.0052^***^	0.000029^***^
	(0.0113)	(0.000288)	(0.0113)	(0.0000041)	(0.00029)	(0.0000040)	(0.0000018)	(0.00029)	(0.0000017)
*N*	232	232	232	176	176	176	176	176	176
*LR test* (*p*–value)	0.000	0.000	0.000	0.000	0.000	0.000	0.000	0.000	0.000

^***, **, *^Indicate significant at the 1, 5, and 10% levels, respectively.

*Z or T-value are in parentheses. The sample period is from 2010 to 2017. Column (1), (2), (3) are the estimated results based on high-income sample group, Column (4), (5), (6), (7), (8), (9) are the estimated results based on low-income sample group*.

Firstly, in column (1), the estimated value of regression coefficient of air pollution on happiness is −0.439, which is significant at 1% significance level. This shows the first step of mediating effect test is passed. Secondly, the estimated value of the regression coefficient of air pollution on physical health is not significant in column (2). Therefore, the significance of the estimated value of the regression coefficient of air pollution on physical health in model (3) should be further tested. Thirdly, in column (3), the estimated regression coefficient of air pollution on health is still not significant. From the above, it proves that there is no mediating effect of physical health on the impact of air pollution on happiness.

Next, the mediating effect of physical health on the impact of air pollution on depression is also tested. Specifically, first, in column (4), the estimated value of regression coefficient of air pollution on depression is −0.0000469, which is significant at the 10% significance level. This means it passes the first step of mediating effect test. Second, in column (5), the estimated value of the regression coefficient of air pollution on physical health is not significant. Therefore, the significance of the estimated value of the regression coefficient of air pollution on physical health in model (6) should be further tested. Third, in column (6), the estimated value of regression coefficient of air pollution on physical health is 0.00172, which is significant at the 1% significance level. From the above, the bootstrap method is continually used to further test the mediating effect of physical health. The results are shown in Table 13.

**Table 13:** Bootstrap test of the mediating effect of physical health in the impact of air pollution on depression.

	**Observed**	**Bootstrap**	***Z***	***P*** **> *z***	**Normal-based**	
	**Coef**.	**Std. Err**.			**[95% Conf. Interval]**	
Indirect effect	−0.00017	0.00008	−2.11	**0.034**	−0.00033	−0.00001
Direct effect	0.00080	0.00018	4.51	**0.000**	0.00045	0.00115

Table 13 shows that the mediating effect of physical health on the impact of air pollution on depression is −0.00017, which is significant under 5%. This shows that physical health plays a mediating role in the impact of air pollution on depression. In addition, the direct effect of air pollution on depression is 0.0008, which is significant at 1% significance level. From the above, physical health has a partial mediating effect on the impact of air pollution on depression, and it may has other mediating effects on the impact of air pollution on depression.

The above process was repeated to test results of the mediating effect of physical health between air pollution and anxiety are also shown. The results are shown in Table 14. It can be seen that physical health has a partial mediating effect on the impact of air pollution on anxiety, and it may has other mediating effects on the impact of air pollution on anxiety.

**Table 14:** Bootstrap test of the mediating effect of body health in the impact of air pollution on anxiety.

	**Observed**	**Bootstrap**	***Z***	***P*** **>** ***z***	**Normal-based**	
	**Coef**.	**Std. Err**.			**[95% Conf. Interval]**	
Indirect effect	−0.00022	0.00009	−2.42	**0.016**	−0.00039	−0.00004
Direct effect	0.00038	0.00015	2.52	**0.012**	0.00008	0.00067

In conclusion, the above results show that the mediating effect of physical health varies with the type of mental health. Specifically, physical health does not play a mediating role in the impact of air pollution on happiness; whereas it has a significant partial mediating effect on the impact of air pollution on depression and anxiety.

## Discussion

In this section, we mainly discuss the comparison between the research in this paper and existing literature, and the reasons behind some of the findings. In general, the conclusions of our study are similar to those of previous studies. Based on the above results, air pollution has no significant effect on happiness and anxiety but has a positive effect on depression when income levels are considered. This confirms the assertation of Zhang et al. that air pollution has increased the risk of depression of residents, but had no significant impact on long-term life satisfaction (24). In addition, the results are in line with Zu et al. They suggest that the heterogeneous impact of air pollution on the comprehensive value of mental health is decided by income (66), which add further insight. First, there is income heterogeneity in the impact of air pollution on happiness, which reaffirm by Maslow's Hierarchy of Needs Theory. Specifically, people with higher income generally have higher demand for environmental quality because their basic material needs have been met. Therefore, air pollution will inhibit happiness significantly. However, this is contraditory to low-income countries where people usually pay more attention to material requirements rather than environmental problems; that is, their happiness can be improved with the improvement of the material living standard. Therefore, air pollution in low-income countries does not have a significant effect on happiness.

Second, the result on income heterogeneity in the impact of air pollution on depression and anxiety also advanced understanding. Studies have shown that developed countries with high-income have increased their defense expenditure against air pollution; whereas low-income countries have different investment willingness on the protective instruments production from private sector (67–69). The results in the study reenforce the notion that people are more prone to depression and anxiety. In other words, air pollution in low-income countries may significantly increase the prevalence of depression and anxiety.

Third, the result of education levels on the effects of air pollution on mental health is interesting to note. It appears that only in high-income countries, air pollution has a significant inhibitory effect on happiness for high educated groups, but not for low-educated ones. This may be explained that people with high-level education would have better ability to obtain information in high-income countries; besides, they would have deeper subjective feelings about air pollution with the increase of environmental knowledge (4). Therefore, air pollution in high-income countries with high education level has a significant negative impact on happiness. In addition, studies have shown that the origin of depression and anxiety are income and economy (20, 21). Therefore, in low-income countries, education levels do not have significant influence on depression and anxiety. Forth, under different income levels with different population densities, the effects of air pollution on mental health are also heterogeneous. First, in high-income countries, air pollution has a significant inhibitory effect on happiness in high-density samples, but not in low-density samples. This may be explained that countries with high density may lead to congestion and resource stress, thus accelerating its inhibiting effect of air pollution on happiness; whereas low density ones can dilute the influence of air pollution on happiness. Therefore, under the condition of high-income level with high population density, air pollution has a significant impact on happiness.

Fifth, the effect of air pollution on depression is significant in low-income with high-density countries, but surprisely not in low-income with low-density countries. This means low-density is critical on reducing the effect of air pollution related depression. Generally speaking, interpersonal communication can help people dispel disappointment, loneliness, and helplessness. Besides, low-income countries with high-density give people more advantages of frequently interpersonal communication; namely, they can get help easily when they are in trouble. For example, they can release some depression to some extent by talking with others when they face air pollution. However, in low-income countries with a low density population, people are living far away, and they may find it difficult to find someone to deal with the depression.

Sixth, the impact of air pollution on the occurrence of anxiety is greater in low-income countries with higher population density. This may be explained that people in low-income countries are more vulnerable to risk and face greater pressure to pay for out-of-pocket health care. When people are facing up to air pollution, they become worry too much about their family safety, as well as their future health. In addition, people may have stronger subjective concern about the harm of air pollution in countries with greater population density. Therefore, in low-income countries, the impact of air pollution on anxiety is significantly positive in different population density countries, as well as the impact in high population density countries is greater than lower ones.

The mediating effect of physical health on the effect of air pollution on different types of mental health is heterogeneous. There is no mediating effect of physical health on the impact of air pollution on happiness in high-income countries. However, physical health has a partial mediating effect of air pollution on depression and anxiety in low-income countries. This may be based on two aspects: on the one hand, high-income people are less likely to be exposed to environmental pollution (67), so the damage to health is not a concern. On the other hand, the health risks of high-income people exposed to air pollution are relatively low because their medical conditions are good (68).

## Conclusions

This paper examined the heterogeneous effects of air pollution on mental health from income, education, indensity, and physical health perspectives. The key findings are summarized as below.

Firstly, there is a heterogeneous impact of air pollution on different types of mental health—happiness, depression, and anxiety. Air pollution has a positive effect on depression; however, the significant impact of air pollution on happiness, and anxiety is decided by income levels. Secondly, income levels pose heterogeneous impact of air pollution on different types of mental health. On one hand, the negative effect of air pollution on happiness is significant only in high-income countries; On the other hand, under different income levels, the heterogeneous impacts of air pollution on different types of mental health are mainly demonstrated in the two factors of education level and population density. Thirdly, the mediating role of physical health in the impact of air pollution on different types of mental health is heterogeneous. To be specific, the mediating role of physical health only significant in the impact of air pollution on the depression and anxiety of low-income groups. In summary, air polution has impact on different mental health, the impact is heterogenous according to primarily inome level, then education level, population density and physical health. The impacts of air pollution contributes to mental health, but the level of impact level differs in terms of happniess, depression, and anxiety. This suggests that a coordinated systemic approach is needed to take mental health issue with duly consideration of air polution and other specific context and conditions.

The results have several implication for policy making in order to to improve mental health. It must be recognized by the relevant public health departments in various countries that air pulution has an impact on different types of mental health. Then, mental health issue should be mainstreamed into governmental and non-governmental policies and programs, parcicularly for low income and high population density countries, such as narrowing the gap among regions for investment of environmental pollution treatment and their regulation standards (69), effectively increasing the income of low-income residents through transferring payment and financial subsidies. Income is not the only factor contributing to mental health, hence economy development and financial support shall not be the only intenvening mechanism. To improve mental health, not only the health sector, but also other sectors, such as education, energy, transport, environment, housing, and welfare, etc., should coordinate to improve the critical conditions affecting mental health. Furthermore, the awareness of environmental protection and improvement should be gradually raised for low-income groups and residents in rural areas, and their enthusiasm of participating in environmental protection should also be increased, i.e., promoting and encouraging residents to buy environment-friendly products through energy-saving subsidies. Most importantly, governments need to encourage businesses to use green and renewable energy sources to reduce air pollution through energy-saving subsidies or providing finance support (70, 71).

Despite the effort to ensure the reliabilty and validity and the rigor of data analysis, this paper still has some limitations. First, the study is based on 51 countries that the findings may not applicable to other countries where the country profile is different. Secondly, categorizing the archieval data and defining the criteria from various data sources are subjective in nature, which may have unconcious bias. Thirdly, although the panel Tobit model is robust fo this type of analysis, it has its own limtations that need to be aware of.

This study can be taken further in the future by examining each specific factor—e.g., national economic variables, meteorological conditions, individual, and social demographic characteristics. Among them, national economic variables denote national investment in environmental governance, green space coverage, etc; regional meteorological conditions refer to factors such as sunshine duration, average wind speed, rainfall, relative humidity and average temperature; and the demographic characteristics of individuals and society are related with age, gender, and working status. Secondly, there may be spatial spillover effect in air pollution, but its spatial correlation is not mentioned in this paper. In fact, air is flowing, and air pollution in a country is easy to spill over the neighboring countries. Therefore, the spatial correlation elements can be added to empirical study in the future. Thirdly, the dynamic impact and its efficiency of air pollution on mental health are not taken into account. Future research can distinguish the effects of air pollution on mental health between long-term and short-term. Lastly, but not the least, a case based approach to examine the country specific policy and intervening mechanism effectiveness could provide useful practice or lessons to be learnt in improving air polution and mental health.

## Data Availability Statement

The original contributions presented in the study are included in the article/supplementary material, further inquiries can be directed to the corresponding author/s.

## Author Contributions

QH and YF conceived and designed the study and supervised the study. MX provided guidance and approved the final manuscript. All authors contributed and discussed about the idea of the study and contributed to manuscript writing and editing.

## Funding

This research was funded by the Education Special Project of National Social Science Fund of China [grant number BIA180197]; Horizontal Project of Zhongda Health Limited Company in Hunan Province [grant number: HYW-2019-003].

## Conflict of Interest

The authors declare that the research was conducted in the absence of any commercial or financial relationships that could be construed as a potential conflict of interest.

## Publisher's Note

All claims expressed in this article are solely those of the authors and do not necessarily represent those of their affiliated organizations, or those of the publisher, the editors and the reviewers. Any product that may be evaluated in this article, or claim that may be made by its manufacturer, is not guaranteed or endorsed by the publisher.
